# Behavioral classification of low‐frequency acceleration and temperature data from a free‐ranging small mammal

**DOI:** 10.1002/ece3.4786

**Published:** 2018-12-27

**Authors:** Emily K. Studd, Manuelle Landry‐Cuerrier, Allyson K. Menzies, Stan Boutin, Andrew G. McAdam, Jeffrey E. Lane, Murray M. Humphries

**Affiliations:** ^1^ Department of Natural Resource Sciences McGill University Sainte‐Anne‐de‐Bellevue Quebec Canada; ^2^ Department of Biological Sciences University of Alberta Edmonton Alberta Canada; ^3^ Department of Integrative Biology University of Guelph Guelph Ontario Canada; ^4^ Department of Biology University of Saskatchewan Saskatoon Saskatchewan Canada

**Keywords:** accelerometer, behavioral classification, decision tree, methods, nest, random forest, red squirrel

## Abstract

The miniaturization and affordability of new technology is driving a biologging revolution in wildlife ecology with use of animal‐borne data logging devices. Among many new biologging technologies, accelerometers are emerging as key tools for continuously recording animal behavior. Yet a critical, but under‐acknowledged consideration in biologging is the trade‐off between sampling rate and sampling duration, created by battery‐ (or memory‐) related sampling constraints. This is especially acute among small animals, causing most researchers to sample at high rates for very limited durations. Here, we show that high accuracy in behavioral classification is achievable when pairing low‐frequency acceleration recordings with temperature. We conducted 84 hr of direct behavioral observations on 67 free‐ranging red squirrels (200–300 g) that were fitted with accelerometers (2 g) recording tri‐axial acceleration and temperature at 1 Hz. We then used a random forest algorithm and a manually created decision tree, with variable sampling window lengths, to associate observed behavior with logger recorded acceleration and temperature. Finally, we assessed the accuracy of these different classifications using an additional 60 hr of behavioral observations, not used in the initial classification. The accuracy of the manually created decision tree classification using observational data varied from 70.6% to 91.6% depending on the complexity of the tree, with increasing accuracy as complexity decreased. Short duration behavior like running had lower accuracy than long‐duration behavior like feeding. The random forest algorithm offered similarly high overall accuracy, but the manual decision tree afforded the flexibility to create a hierarchical tree, and to adjust sampling window length for behavioral states with varying durations. Low frequency biologging of acceleration and temperature allows accurate behavioral classification of small animals over multi‐month sampling durations. Nevertheless, low sampling rates impose several important limitations, especially related to assessing the classification accuracy of short duration behavior.

## 1 INTRODUCTION

In recent years, accelerometers have become an important tool in ecology, initially used in marine ecosystems where direct observations are difficult and the need for a device that records what cannot be observed was necessary (Brown, Kays, Wikelski, Wilson, & Klimley, 2013; Yoda et al., 1999). Since then, there has been a slow integration of these dataloggers by terrestrial wildlife biologists to aid in the quantification of energy expenditure, activity levels, and animal behavior (Gleiss, Wilson, & Shepard, 2011; Wilson, Shepard, & Liebsch, 2008). An exciting opportunity afforded by biologgers is the potential to document how the behavior of free‐ranging animals, including their time budgets (McClintock, Russell, Matthiopoulos, & King, 2013), movement rates (Heurich et al., 2014), and occurrence of specific acts like predation (Williams et al., 2014), mating (Whitney, Pratt, Pratt, & Carrier, 2010), specialized feeding (Watanabe & Takahashi, 2013), or refuge occupation (Körtner & Geiser, 2000), corresponds with temporal variation in temperature, photoperiod, and resource availability operating over daily (e.g., photoperiod), monthly (e.g., moon phase), annual (e.g., seasons), and multi‐annual time scales. However, constraints related to biologger battery life, memory capacity, and device size generate a trade‐off between sampling rate (frequency of recording) and sampling duration (the recording interval between the start and end of observations). While high sampling rates are attractive because they offer more accurate information at higher temporal resolution, they often require sampling durations that are much shorter than many ecologically important timescales. Small animals that cannot carry large biologgers are most constrained in this way.

The sampling rate versus duration trade‐off is made more extreme with accelerometers by the recommendation that recording frequencies need to be at least twice that of the highest frequency movement of the individual (Brown et al., 2013). For small species, which consequentially have the highest stride frequencies (Bejan, Marden, & Ansell, 2006), this requires a recording frequency between 8 and 100 Hz (Brown et al., 2013). This results in a potential maximum recording longevity in the order of minutes to days, unless sub‐sampling techniques are used (Hammond, Springthorpe, Walsh, & Berg‐Kirkpatrick, 2016). Unfortunately, such short sampling duration severely constrains the forms and extent of temporal variation that can be incorporated into behavioral studies. An alternative method is to extend the sampling duration by reducing the sampling rate. If behavioral classification is possible at recording frequencies of 1 Hz or slower, sampling period could be increased from hours or days to weeks, months, or years, again depending on the size of the tag possible given animal mass. However, the few studies that have directly tested this possibility suggested that low recording frequencies have significantly reduced accuracy when using current classification methods (Broell et al., 2013; Pagano et al., 2017; Wang et al., 2015).

For species‐specific calibrations, a variety of methods have been proposed for the conversion of raw acceleration values into behavioral states (Bidder et al., 2014; Collins et al., 2015; Nathan et al., 2012). Many methods use supervised machine learning algorithms and among the most popular methods is the random forest algorithm (Breiman, 2001), which uses known data to generate numerous decision trees and calculates the overall relative importance of each variable with which it was provided (Graf et al., 2015). The black box nature and data specificity of these methods makes it difficult for researchers to assess the logic, validity, and accuracy of applying classification schemes developed with training data to new applications lacking training data (Bidder et al., 2014; McClune et al., 2014). As alternatives to such methods, arguments have been raised for more simplified analytical techniques such as manually creating decision trees (Collins et al., 2015). Although more time consuming, the hands‐on nature of this approach, likely results in a more comprehensible classification that should be more easily transferable to new applications.

**Figure 1:**
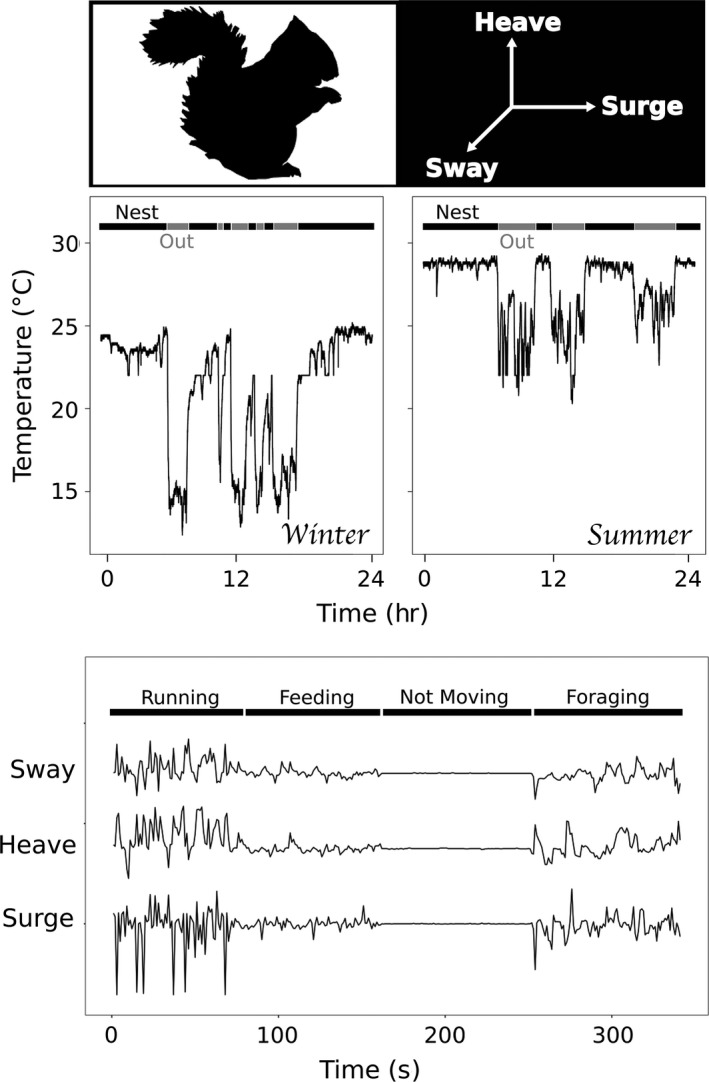
Example of temperature and acceleration biologger data on red squirrels demonstrating the distinct signatures of different behavioral states. This includes in (black bars) and out (gray bars) of the nest in the temperature data during both winter and summer, and running, feeding, not moving, and foraging signatures in acceleration data

In addition to acceleration, many accelerometer devices designed for wildlife research are equipped with built‐in temperature loggers (Figure 1). Although often overlooked and under used, recorded temperature can provide important supplementary information about an individual and its thermal micro‐environment. When attached externally to an animal, the temperature recorded is often intermediate between body temperature and the ambient temperature of the environment immediately surrounding the individual (Osgodd & Weigl, 1972; Studd, Boutin, McAdam, & Humphries, 2016; Tremblay, Cherel, Oremus, Tveraa, & Chastel, 2003). This temperature intermediacy likely accounts for their rarity of use; collar temperature is not a reliable measure of body temperature or air temperature (Audet & Thomas, 1996; van Beest, Moorter, & Milner, 2012). However, depending on the ecology of the species and which of these two temperatures vary more, collar temperature can be used to monitor thermal exposure (Osgodd & Weigl, 1972; Kanda, Fuller, & Friedland, 2009) or heterothermic fluctuations indicative of torpor expression or hibernation (Lazerte & Kramer, 2016). Most pertinent here, collar temperature likely offers useful information about behavioral state, as it tends to more closely approximate the body temperature of inactive animals confined in small spaces (e.g., thermal refuges) and to more closely approximate the air temperature experienced by active animals fully exposed to ambient conditions (Körtner & Geiser, 2000; Messier, Taylor, & Ramsay, 1994; Murray & Smith, 2012; Olson et al., 2017; Wassmer & Refinetti, 2016).

An ideal candidate for investigating the potential for low frequency recordings is the North American red squirrel (*Tamiasciurus hudsonicus*), as their small size (~250 g) drastically restricts potential battery life of biologging devices. This diurnal homeotherm uses insulated nests during rest periods, and remains active year‐round (Guillemette et al., 2009; Humphries et al., 2005). In the northern boreal forest, they larder hoard resources every autumn to sustain activity and reproduction during the winter, resulting in large variation in activity and energy expenditure throughout the year (Fletcher et al., 2013; Humphries et al., 2005; McAdam, Boutin, Sykes, & Humphries, 2007). Due to the fact that they are diurnal, and actively defend small (0.3 ha) territories (LaMontagne et al., 2013; Smith, 1968), individuals are relatively easy to capture and observe in the wild.

Here, we used a combination of low‐frequency (1 Hz) acceleration and temperature recordings on free‐ranging red squirrels to develop methods for biologger‐based behavioral classification. Our main objective was to determine whether accurate classifications can be achieved using low‐frequency accelerometer and temperature recordings from a small animal, and determine what modifications to common recommendations for behavioral classification methods of accelerometer data would be needed. The first part of our analysis develops a method that integrates temperature data into the behavioral classification allowing for identification of whether or not the individual is in a thermal refuge or not. The second part of our analysis explores different analytical approaches to the accelerometer classification to determine best practices for low‐frequency data. We initially complete the classification using the commonly used random forest approach, and use this to explore how selection of sample window size can affect both the accuracy of the classification and structure of the resulting behavioral dataset. Using this information on sample window size, we then manually create a hierarchical decision tree that starts with the broadest classification of behavior (2‐behavior: not moving, moving) and then expands in detail with each subsequent branch until a 6‐behavior classification is reached (see Table 1 for description of each stage). This approach creates a tree that can be easily clipped for the level of detail that is desired for different ecological questions. Finally, we compare the accuracy of the classification using the random forest algorithm to that of our manually created decision tree. This study demonstrates that manually created decision trees give a greater level of understanding and control over the classification, and allows adjustment of sampling windows to the characteristics of naturally occurring behavior. We show how to achieve accurate behavioral classifications on free‐ranging small animals using low‐frequency accelerometer recordings and conclude by highlighting some of the difficulties that may be faced when trying to implement this method on other free‐ranging species, as well as how best to overcome them.

**Table 1:** Definitions of each behavioral category used in each step of the hierarchical decision tree completed in this study. Table illustrates how each subsequent behavioral state is nested within a category of a less complex tree.

2‐Behavior		4‐Behavior		5‐Behavior		6‐Behavior	
Category	Definition	Category	Definition	Category	Definition	Category	Definition
Out of nest	Outside a thermal refuge	Moving	Outside a thermal refuge and some part of the animal is moving	Traveling	Animal is moving in space at either a slow or fast locomotion state	Foraging	Slow locomotion consistent with searching for and collecting food
						Running	Fast locomotion consisting of more than 1 stride at a time
				Feeding	Not moving in space but body is moving with the handling and ingesting of food		
		Not moving	Outside a thermal refuge and no part of the animal is moving except for breathing				
In nest	Inside a thermal refuge	Moving	Inside a thermal refuge and some part of the animal is moving				
		Not moving	Inside a thermal refuge and no part of the animal is moving except for breathing				

## 2 MATERIALS AND METHODS

### 2.1 Study site and species

Between February and October 2014, we studied free‐ranging North American red squirrels in southwestern Yukon (61°N, 138°W), a population that has been part of a long‐term study since 1987 (McAdam et al., 2007). Male squirrels were trapped on their territories using Tomahawk live traps baited with peanut butter, and fitted with a collar (total weight = 8 g) combining a ventrally mounted VHF radio‐transmitter (model PD‐2C, 4 g [1.7% of body mass], Holohil Systems Limited, Carp, ON, Canada) and a dorsally mounted tri‐axial accelerometer (model Axy2, 4 g [1.7% of body mass], Technosmart Europe). Accelerometers were set to record forces between −8 and 8 g_force_ at 1 Hz. Collars were constructed in the field on day of deployment (see Supporting Information Appendix S1: Section 1.1). Once collared, squirrels were released and remained free‐ranging, including during focal observations (see below), until they were recaptured an average of 22 days (range 5–65) later and collars were removed. During 2014, we deployed 37 accelerometers on 20 individual red squirrels in winter (February) and mating season (March), 25 accelerometers on 18 individuals in summer, and 30 accelerometers on 30 individuals in autumn for a total of 1,924 days of recordings.

### 2.2 Behavioral observations and scoring

We used two methods to record instantaneous behavioral states of free‐ranging red squirrels. In winter 2014, we located individuals using VHF and continuously recorded behavior for 2 min using an application built for iPod touch (see Supporting Information Appendix S1: Section 1.2). Six behavioral states were recorded: feeding, not moving, in nest, running, slow travel, and stationary movement (defined as not traveling but still moving: e.g., grooming, vocalizing).

In autumn 2014, in addition to recording continuous behavior on the iPod app, we located individuals and recorded behavior with a video camera (Sony Handycam HDR‐CX240) for as long as the individual was visible. Videos were watched by two observers and scored in real time, recording the start and end time of each behavior. Autumn behavior was categorized as: caching, clipping cones, digging, feeding, grooming, running, slow travel, and vocalizing. For all analyses, observed behavior was then combined into in nest, not moving, feeding, foraging (caching, clipping cones, digging, slow travel), stationary movement (grooming, vocalization), and traveling (see Supporting Information Video S1). Over both winter and autumn deployments, we completed 1,165 two‐minute observations on 20 individuals, and video‐recorded a total of 83.8 hr of direct observation on 27 individuals with videos ranging from 15 s to 12 min in duration.

### 2.3 Adjusting for time errors

Although we made every effort to ensure that accelerometers and time devices used for behavioral observations were synchronized upon deployment, the internal clocks on the different devices did not run precisely at the same rate. This resulted in small deviations, in the order of seconds, that would not be noticed if only a single recording device was used. However, when trying to synchronize and cross‐reference observations recorded by two devices, such as an accelerometer and a focal observation app, these small deviations were significant, especially because most recorded behavior lasted for only a few seconds. We corrected the time on the observations by aligning abrupt changes in acceleration with abrupt changes in observed movement data (resting to traveling, and vice versa) on the two devices (see Supporting Information Appendix S1: Section 1.3 for details). We removed from future analysis all squirrels for which there was not an abrupt change in the observed data during a given day (320 of 403 squirrel days), leaving 46 squirrel days (12 individuals; 378 min) from the winter and 37 squirrel days (18 individuals; 326 min) from the autumn observational periods in the analysis.

### 2.4 In nest versus out of nest

Red squirrels spend considerable amounts of time in their nests, during which time they mostly rest. The first stage of our classification was to identify whether or not the individual was in a nest. The accelerometer units recorded temperature in addition to acceleration (Figure 1). Following a similar method used by Studd et al. (2016), we inferred nest‐use based on the concept that the ambient temperature of the local environment (i.e., surrounding the squirrel) is warm and stable when in the nest, and cold and variable when out of the nest. Before analysis, all temperature data were smoothed to filter out erroneous recordings (see Supporting Information Appendix S1: Section 1.4). For each day of recordings (12 p.m.–12 p.m.), we used *k*‐means clustering constrained to two clusters to determine a daily threshold temperature, above which a squirrel was considered to be in the nest (Studd et al., 2016). This threshold is unique to each squirrel and day to account for changes in nest insulation (Guillemette et al., 2009), orientation of collar, and daily ambient temperature. As there are some occasions when squirrels were observed to be active and out of the nest but the temperatures were above the threshold, possibly as a result of the individual sitting in the sun, we imposed an additional constraint using the squirrel's activity levels from the accelerometer data. We assumed that squirrels use nests primarily for resting and, therefore, should not be moving most of the time they are in the nest. Thus, we calculated the proportion of each nest bout that the squirrel was moving versus not moving (using the method below) and reclassified any nest bout where the ratio of moving to not moving was above 1, as being out of the nest.

### 2.5 Moving versus not moving

Prior to all accelerometer analysis, we separated the static acceleration from the dynamic acceleration by applying a running means smoothing function at a window of 91 s. Following methods proposed by Shepard et al. (2008) on selecting appropriate window size for the smoothing, we completed a sensitivity analysis of the window on the estimation of overall dynamic acceleration (see Supporting Information Appendix S1: Section 1.5). Although body orientation and posture can be determined from the static acceleration, we only used the dynamic acceleration (raw acceleration minus smoothed acceleration) for all analysis.

**Figure 2:**
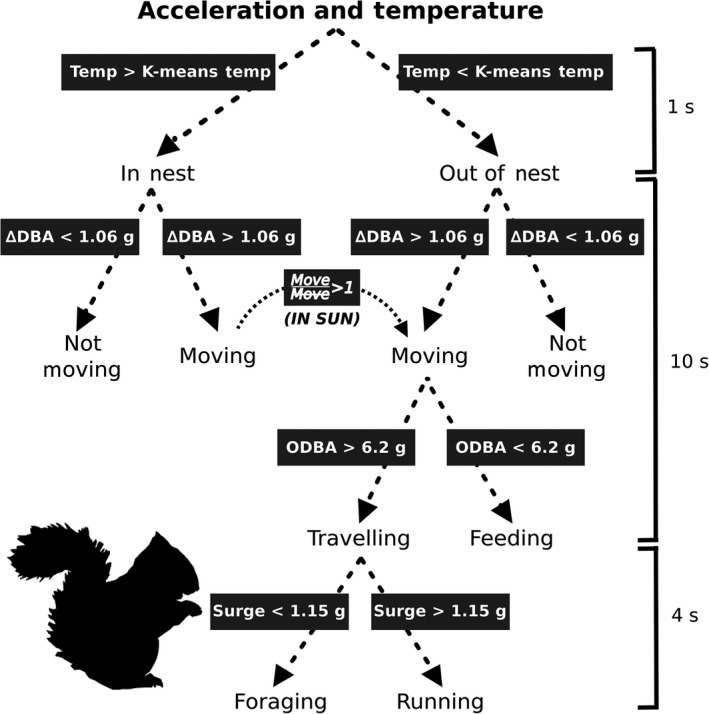
Classification decision tree of behavior from animal‐borne acceleration and temperature biologgers on wild North American red squirrels. Red squirrel use of insulated nests can be identified through temperature signatures while behavioral state can be classified using acceleration. Classification was done at sample windows relevant to the natural duration of each behavior. For example, short duration behavior like running was classified at 4 s sample windows. Values in dark gray are the summary statistics and threshold values (in g_force_) used for each division

The first level of classification of the accelerometer data was to determine when the squirrels were moving or not moving (Figure 2). In most deployments, accelerometers would be turned on, packaged, and then sat on a table for 30 min to 8 hr prior to being deployed. We selected a 1,000 s section during this time from 36 accelerometers (14 winter, 22 autumn deployments), and calculated the delta dynamic body acceleration (ΔDBA), defined as:

$$\Delta \text{DBA}=\sum_{i=1}^{t}\Delta a_{\mathrm{xi}}+\Delta a_{\mathrm{yi}}+\Delta a_{\mathrm{zi}}$$

where the change in dynamic acceleration (Δ*a*) for the surge (*x*), sway (*y*), and heave (*z*) axis is calculated for each recording and then summed across a sample window (*t*). From our data of devices sitting still, we selected the 99.9% quantile of these ΔDBA measurements at a sample window (defined as the no. of consecutive acceleration records over which the statistic is calculated) of 14 s as the threshold (1.06 g_force_; Figure 2), above which the device was considered to be moving and below which to be not moving.

### 2.6 If moving: Feeding versus foraging versus traveling

For the next stage of our hierarchical classification (Figure 2), we took all accelerometer data that indicated periods of movement and divided them into the three most common moving behavioral states (feeding, foraging, and running; 97.4% of observed movement). Using the random forest classification algorithm in R (Svetnik et al., 2003), we tested the degree to which accuracy of classification varied with the chosen sample window (2, 4, 7, 10, 14, 20, and 30 s). For this, we separated the focal observation data into segments of the desired sample windows, identified the most common behavior within these sample windows, calculated their duration, and selected only segments that met the following criteria: 100% of sample window was feeding, at least 75% was foraging with 0% running, or at least 51% was running (as running rarely continuously lasted for more than 4 s). To create our training dataset, we randomly sampled equal numbers of each behavior from this pool of segments. Across each window, we calculated six summary statistics on the dynamic acceleration of each axis (mean, standard deviation, maximum, sum, range, and sum of Δ*a*), the overall dynamic body acceleration (ODBA; sum of the absolute values of dynamic acceleration; Wilson et al., 2006), ΔDBA, minimum Δ*a*, maximum Δ*a*, maximum acceleration, mean pitch, and mean roll using all three axes together for a total of 25 different summary statistics. All statistics were input into the random forest algorithm using 75% of observations for training (growing 2,000 trees), and 25% for calculating the accuracy.

**Figure 3:**
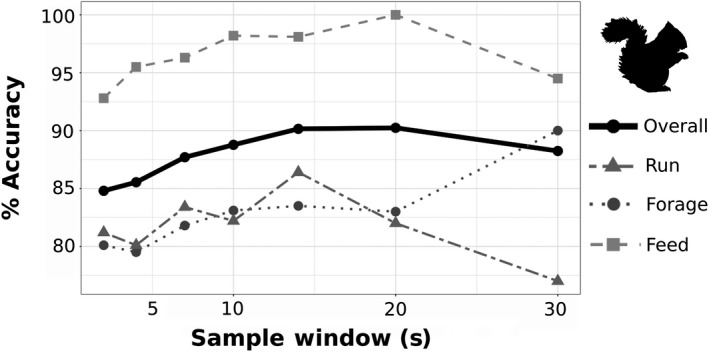
Example of methodology used for determination of threshold values in separating two behavioral states. Histograms of summary statistics were plotted to determine which statistics visually had the clearest distinction between two behavioral states (a). The optimal threshold value was then determined by assessing the accuracy of classification of each known behavior across the selected summary statistic (b). Here, ODBA showed a clear division between red squirrel feeding and traveling (a) and an ODBA value of 6.2 g_force_ produced the highest overall accuracy (92.1%; b)

As an alternative method, we constructed a manual decision tree for classification using R (R Core Team, 2017). Our first division of moving behavior was into two categories: feeding and traveling. We selected a sample window of 10 s; we considered this sample window to be long enough that only the two behavioral states of interest would be relevant (those that naturally occur at that duration or longer). Following rationale suggested by Collins et al. (2015), we initially plotted histograms of all summary statistics for each behavioral category to visually determine which statistic had the clearest division between the two behavioral states (Figure 3). We then ran an optimization calculating the % error of classification of known behavior across a range of values of that statistic to determine a threshold value. This method is easily repeatable for separation of any behavioral states and was used to subsequently separate running from other forms of traveling (foraging), using only the segments of behavior that were correctly classified in the previous division. Since the average duration of running behavior in red squirrels is 4 s (see Section 3), we ran this last division at a 4 s sample window.

### 2.7 Testing overall accuracy

Once our decision tree was built, we tested the accuracy in two ways: (a) at high resolution with the full observational dataset of detailed continuous behavioral observations used in the training as is commonly done in accelerometer calibrations (Bidder et al., 2014), and (b) at lower resolution with a 7 min behavioral observation data set that was collected concurrently during autumn 2014. We chose to test accuracy at two resolutions to explore whether issues with the time alignment may be influencing the accuracy values at the high resolution. These latter observations recorded behavior of each individual squirrel every 30 s for 7 min, as well as the occurrence of critical incidents defined as vocalizations, caching, and new feeding events. From these (*n* = 509), we selected only those 7 min observations where the individual spent 95% of the observations feeding (*n* = 45), or traveling (*n* = 50). For this analysis, traveling was defined as any combination of foraging and running as they always co‐occurred over the course of 7 min. To eliminate issues of time alignment, we selected the inner 5 min of these observational periods and tested whether the most common behavior from the accelerometer classification aligned with the most common behavior during those 5‐min periods. To test accuracy of in/out of the nest for both accuracy measures, we assumed that since squirrels are diurnal and are known to sleep in nests that the majority of each night all squirrels should be in nest. Thus, we randomly selected 400, 15‐s samples between 10 p.m. and 4 a.m. from accelerometers that were deployed during winter (*n* = 200) and autumn 2014 (*n* = 200) and labeled them as in nest. Accuracy was calculated for each step of the hierarchical decision tree (2, 4, 5, and 6‐behavior classifications; Table 1) on a random subsample of 50 observational event for each behavior in the respected classification. We calculated the average accuracy and standard deviation for each behavior by repeating the subsampling process 100 times. Accuracy was calculated for the lower resolution behavioral dataset following the same method with the exception that: (a) each subsampling selected 15 random observational events for each behavioral state due to lower total sample size, and (b) only for the 5‐behavior classification tree due to which behavioral states were recorded at this lower resolution.

### 2.8 Red squirrel seasonal time budgets

We calculated the average time budget per season for red squirrels using all accelerometer recordings used for the calibration. We selected a 10‐day period in each season (winter: February 15–25, *n* = 15; mating: March 10–20, *n* = 9; summer: June 10–20, *n* = 12; autumn: September 5–15, *n* = 24) and included all squirrels that had accelerometer recordings during that time. All recordings were converted to behavior using our decision tree (Figure 2), and the proportion of each day spent doing each behavior was calculated. To test whether time budgets varied with season, we used a MANOVA analysis with a Pillai test in R (Fox & Weisberg, 2011) where the number of seconds per day spent doing each of six the behavioral states were the dependent variables, and season and squirrel id were the explanatory variables.

## 3 RESULTS

### 3.1 In nest versus out of nest

Using a *k*‐means cluster analysis to determine daily threshold temperatures for in/out of the nest and a movement‐based correction, we achieved an overall nest classification accuracy of 91.6 ± 2.5%. The classification had higher accuracy for the out of nest (observed feeding, or traveling) category (93.8 ± 3.4%) than for the in nest category (89.3 ± 3.9%).

### 3.2 Moving and not moving classification (Two behavior level)

Before testing the accuracy of the moving/not moving threshold (ΔDBA = 1.06), we removed all behavior classified as “in nest” from the testing dataset as we did not have visual confirmation of whether the individuals were moving or not while in the nest. On the remaining training data set of known behavior, the threshold had an overall accuracy of 90.3 ± 2.3%. The accuracy of known not moving behavior was 83.9 ± 4.7%, and for known moving behavior was 96.9 ± 2.3%.

### 3.3 Classifying moving behavior

**Figure 4:**
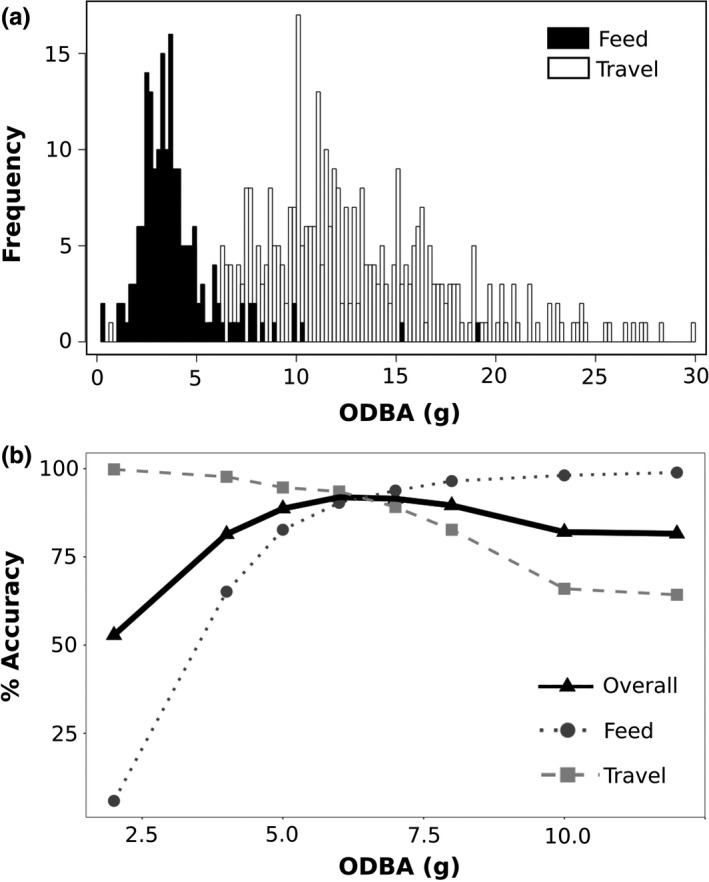
Percent accuracy of random forest algorithm at classifying accelerometer data to known active behavioral states at varying sample windows for red squirrels. Overall accuracy is the mean accuracy of the three behavioral states: running, foraging, and feeding

We tested how different sampling windows influenced classification accuracy of the random forest machine learning algorithm. The overall accuracy increased with increasing sample window size from 84.8% correct classification at 2 s to 90.2% at 20 s before decreasing at the longest window size (Figure 4). The ability to distinguish feeding behavior was consistently above 90% for all sample windows increasing from 2 to 20 s. Foraging and running varied from 77% to 90.0% accuracy. However, using different sample window sizes influenced the average duration of each behavior classified through the analysis (Table 2).

**Table 2:** Average durations in seconds of each behavior common in red squirrels calculated from different classification methods. Winter and autumn durations are tabulated from observations of free‐ranging squirrels during each season (winter: 18 squirrels, 2,328 min; autumn: 27 squirrels, 621 min). These are compared to durations calculated from classified accelerometer data from 6 squirrels (3 winter, 3 autumn) using the random forest method with varying sample sizes of 2–30 s, and a manual decision tree method (DT)

	Observed		Predicted—Random forest							DT
	Winter	Autumn	2	4	7	10	14	20	30	
Feed	45.89	24.03	3.84	20.24	32.1	48.5	54.5	72.49	81.61	57.75
Forage	8.29	10.56	3.22	8.02	13.07	18.43	23.86	32.69	58.33	19.01
Run	5.2	3.77	3.78	11.82	19.74	28.98	42.94	66.05	60.47	7.44

The first step of the manual decision tree method was to separate feeding behavior (consumption of food) from traveling (foraging and running). We identified that ODBA provided the clearest division between the two categories. Optimization across a range of ODBA values produced the highest classification accuracy (92.1%) at a threshold of 6.2 g_force_ (Figure 3). Since the natural average duration of running was 4 s (Table 2), we selected that as our sample window to classify running from other traveling (foraging). We identified that the maximum value of the surge axis had the greatest distinction between the two behavioral states, although there was considerable overlap, with a threshold value of 1.15 g_force_ providing the highest overall accuracy of 71.9%, with individual accuracies of 78.0% and 65.9% of distinguishing foraging and running among the observations that had been classified as non‐feeding behavior in the previous step of the decision tree (Figure 2).

### 3.4 Overall accuracy of decision tree

Accuracy decreased with increasing complexity of the decision tree where the highest accuracy occurred at the 2‐behavior tree (91.6 ± 2.5%), and lowest accuracy at the 6‐behavior tree (70.6 ± 2.3%; Table 3). For individual behavioral states, the accuracy was high for in nest (89.4 ± 4.2%), feed (86.3 ± 4.3%), and out of nest not moving (83.9 ± 4.7%), and low for foraging (66.4 ± 5.9%) and running (26.8 ± 5.8%). For these last two behavioral states, most error was associated with misclassification of running as foraging and vice versa as the combined category of the two behavioral states (traveling) had high accuracy of classification (89.4 ± 3.6%). Using an independent data set of 5 min observational periods, we were able to test the accuracy of the classification of feeding, traveling (foraging and running), and in nest behavior. The overall accuracy was 96.4 ± 1.7%, with individual accuracies of 97.8 ± 3.1% for feeding, 91.5 ± 6.9% for in nest, and 100 ± 0.0% for traveling.

**Table 3:** Mean percent accuracy of the manually created decision tree at correctly classifying each behavioral state in four trees of increasing complexity. Mean accuracy is calculated over 100 subsampling events of observational data (50 observations per behavioral state). There is no observational data of whether red squirrels were moving or not moving while in the nest so those two categories were combined as “In nest” for the 4, 5, and 6‐behavior classification trees

2‐Behavior		4‐Behavior		5‐Behavior		6‐Behavior	
Category	Mean ± *SD*	Category	Mean ± *SD*	Category	Mean ± *SD*	Category	Mean ± *SD*
Out of nest	93.8 ± 3.4%	Moving	96.9 ± 2.3%	Feeding	86.7 ± 4.0%	Feeding	86.3 ± 4.3%
				Traveling	89.4 ± 3.6%	Foraging	66.4 ± 5.9%
						Running	26.8 ± 5.8%
		Not moving	83.9 ± 4.7%	Not moving	84.2 ± 4.6%	Not moving	83.9 ± 4.7%
In nest	89.3 ± 3.9%	In nest	90.0 ± 4.1%	In nest	89.8 ± 4.2%	In nest	89.4 ± 4.2%
Total	91.6 ± 2.5%	Total	90.3 ± 2.3%	Total	87.5 ± 1.9%	Total	70.6 ± 2.3%

### 3.5 Seasonal time budgets

**Figure 5:**
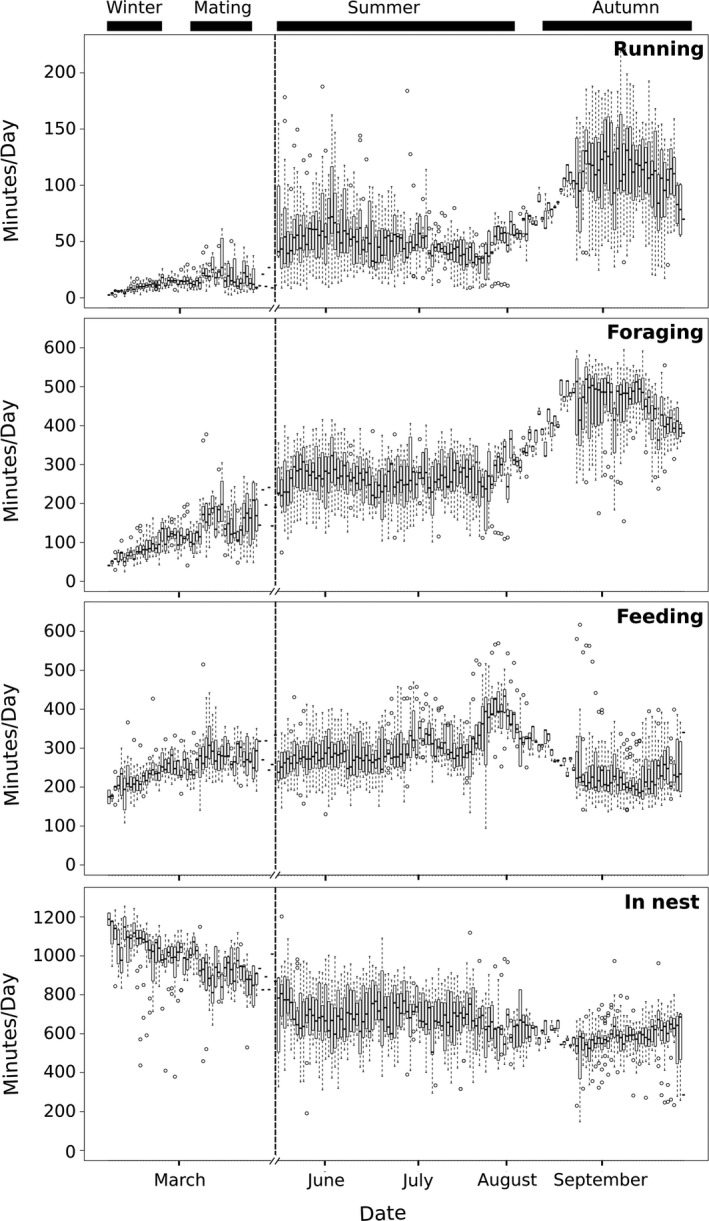
Time red squirrels spent each day from late winter to late autumn doing each of the four main behavioral states: running, foraging, feeding and in nest. Each box represents the interquartile range of all individuals as calculated from classified accelerometer data using a manual decision tree classification. The dotted line signifies a break in the time line when no accelerometers were deployed

Red squirrels adjusted daily time budgets between seasons (MANOVA Pillai = 1.25, *F* = 70.0, *df *= 15 and 1,467, *p* < 0.001), spending considerably more time in the nest not moving during winter (64.5 ± 0.7% of 24 hr) and mating season (56.5 ± 1.2%) than in summer (43.9 ± 0.8%) and autumn (36.4 ± 0.3%). Time spent foraging and running was the greatest during the autumn hoarding period (forage = 32.25 ± 0.3%, running = 7.9 ± 0.2%) and least during winter (forage = 5.7 ± 0.1%, running = 0.7 ± 0.0%), with intermediate amounts during summer (forage = 17.7 ± 0.3%, running = 3.6 ± 0.2%) and mating (forage = 10.9 ± 0.6%, running = 1.3 ± 0.7%). The amount of time spent feeding (autumn = 15.0 ± 0.2%, mating = 20.2 ± 0.6%, summer = 19.1 ± 0.3%, winter = 15.7 ± 0.02%), in nest moving (autumn = 4.1 ± 0.1%, mating = 6.1 ± 0.2%, summer = 5.5 ± 0.2%, winter = 7.6 ± 0.1%), and not moving (autumn = 4.4 ± 0.3%, mating = 4.8 ± 0.5%, summer = 10.3 ± 0.6%, winter = 5.7 ± 0.6%) were the most consistent behavioral states between seasons (Figure 5). These seasonal time budget differences were expressed consistently by most individuals across most seasons.

## 4 DISCUSSION

We demonstrate that accurate behavioral calibrations are achievable using low‐frequency accelerometer recordings on free‐ranging species with a decision tree methodology that is simple to use and easy to interpret. Our classification of 1 Hz acceleration and temperature recordings of red squirrels into six behavioral categories had an accuracy of 70.6%. However, classifying into five behavioral categories had a much improved accuracy of 87.5% in matching high resolution observational data and 96.4% accuracy in matching to the general behavioral state during 5 min visual observations. This was the first terrestrial study, to our knowledge, that integrates acceleration with temperature, producing information on behavioral state as well as whether that behavioral state is expressed inside or outside of a thermal refuge. Using this calibration, we were able to produce the first seasonal time budgets for North American red squirrels, showing that there are substantial changes in daily behavior between seasons (Figure 5).

Here, we showed that the standard accelerometry practice of high frequency recording may not be as necessary as previously suggested. Our calibration of 1 Hz acceleration data yielded high overall accuracy while allowing continuous recordings on red squirrels for up to 2 months per deployment. This contrasts alternative methods of increasing sampling duration of these devices through non‐continuous sampling regimes. For example, on chipmunks, Hammond et al. (2016) recorded at a commonly recommended 20 Hz which required a sampling regime of 10 s every 15 min in order to achieve a 4.5‐day sampling period. If a continuous recording regime had been used, the maximum sampling period would have been just over one hour. While our study design (exclusive reliance on low frequency sampling) does not permit direct comparison of accuracies that could have been obtained with higher sampling rates, we can assess this indirectly by examining the classification accuracy of behavioral states of variable duration (see recommendation 2 below). However, future research on the direct comparison of sampling rate is warranted by subsampling a higher frequency recording while allowing for variation in sample window size with each new sampling rate (see recommendation 3 below). Currently, all comparative studies that have been completed maintain the same sample window size for all recording frequencies (Pagano et al., 2017; Wang et al., 2015) which may be driving the sudden and drastic decrease in accuracy seen at low frequency recordings (Figure 4). Despite this, the accuracies that we achieved here (70.6%–91.6%) were comparable to other studies which sampled at much higher rates (3.3–40 Hz) with accuracies ranging from 75% to 98% (Bidder et al., 2014; Hammond et al., 2016; McClune et al., 2014; Nathan et al., 2012).

Our study is one of few that has completed a calibration using free‐ranging individuals. Although many calibrations use captive animals or surrogate species for training data (Campbell, Gao, Bidder, Hunter, & Franklin, 2013), Pagano et al. (2017) showed that highest accuracy is achieved using free‐ranging individuals of the same species of interest. We followed this advice by incorporating observations from both low activity (winter) and high activity (autumn) seasons for training, attempting to incorporate the full range of potential movements that red squirrels might express in the calibration. Although specific behavioral states may change between seasons, at the broad behavioral categories that we were using, there was no evidence that there were distinct enough seasonal differences to merit a separate calibration for each season, but future research could explore this in more detail. The one aspect of the calibration that may be susceptible to seasonality is the use of temperature for determining in and out of the nest, where the efficacy depends on the nest temperature being distinctly warmer than ambient air temperature (Osgodd & Weigl, 1972). The population of red squirrels used in this study lives in a climate where this is always the case, and even in summer, we found that you could clearly distinguish between in and out of the nest (see Figure 1 for example), but this may not be the case in all studies. Despite the high accuracy that we achieved, we would like to highlight a couple issues that were encountered with both low frequency recordings and working on free‐ranging individuals that may be common to others who follow a similar methodology.

First, when recording at a lower frequency than the stride frequency of the species, some commonly used and recommended analytical techniques (spectral analysis, orientation; Brown et al., 2013) may become less applicable. At 1 Hz, the data recorded is a snapshot of acceleration values from each movement type. This means that it will not always record the peak acceleration that was reached but some value along the wave of accelerations experienced during each stride. Second, no two time devices will record time at precisely the same rate due to variation in crystal oscillating frequency in each time device, which is influenced by general noise, voltage change, temperature, and aging of the clock (Syed & Heinemann, 2006). When there is a need to precisely align instances recorded on two devices, for example to calibrate one observation method via another using instantaneous observations recorded every second or less, then small time offsets become noticeable and problematic. Until behavior and accelerometer data can be collected over a network with a shared clock, studies on free‐ranging animals fitted with non‐networked, store‐on‐board biologgers will face this problem (Gaylord & Sanchez, 2014).

Although these two issues do make field‐based calibrations of low‐frequency acceleration more difficult, they do not preclude detailed time budget classification and an overall assessment of classification accuracy. We conclude the paper with some recommendations for behavioral classifications using low‐frequency acceleration, applicable to a research context in which the priority is to accurately classify major behavioral states, recorded continuously, across a sampling period of maximum length.

1. Behavioral observations used for calibrations should be continuous and as long in duration as is possible for the study species: More stark transitions between behavioral states (traveling to not moving and vice versa) within each observational period makes it easier to accurately align events recorded on both devices. That being said, the feasibility of long duration, continuous observation sampling varies by species. Although red squirrels are relatively easy to observe, their small size, arboreality, and rapid movement, all within a three‐dimensional visual obstructed forested landscape makes long continuous observations challenging to obtain. As a result, we had to remove 80% of observational periods (320 of 403 squirrel days) from the analysis because in too many instances we did not observe enough major transitions within a single continuous bout to accurately and objectively align time as recorded by the accelerometer and the observer.
2. Select behavioral states that naturally occur at durations longer than both the recording frequency, and the error in the time alignment: Longer duration behavioral states provide the opportunity to select the middle segment of each occurrence, thereby eliminating the chance of working with mislabeled accelerometer data from misalignment. If classifying a behavior that typically lasts for 2 s, using a 1 Hz sampling rate with a 2 s error in alignment, the likelihood that the labeled segment will include the matching acceleration is only ~30%. We found that short duration behavior had the lowest accuracy (26.8%) when testing on observational data aligned to the second, a pattern that is common to other studies (Pagano et al., 2017). We used a lower resolution observational data set (5‐min) to test whether the low accuracy is likely resulting from misalignment of time. If the inaccuracies are the result of poor ability of the decision tree than we would expect that accuracies would be similar between the low and high‐resolution datasets, while if the errors are stemming from time alignment then we would expect higher accuracy at the lower resolution when time misalignment has minimal effects. We found that accuracy of lower resolution data was ~10% greater than the higher resolution data, suggesting that our estimate of classification accuracy for short behavior is likely more conservative than is actually the case. Though this is vaguely reassuring, accurate classification (and assessment of classification accuracy) becomes an increasingly intractable challenge as behavioral duration begins to approximate sampling rate. The trade‐off between sampling rate and sampling duration dictated by the accelerometer manifests as the same trade‐off affecting the behavioral classification. Extending the sampling duration by reducing the sampling frequency inevitably compromises detection of behavioral events confined to very short time intervals. Thus, researchers will need to set expectations to either accurately documenting fine scale behavior continuously at millisecond sampling rates, or accurately documenting long‐term behavior continuously over months and years, as likely both, at the same time, with the same device will not be possible.
3. Create classifications using a manual decision tree: There are two key benefits to this approach over a machine learning algorithm. First, creation of hierarchical decision trees become possible, such that classification can be performed at multiple levels of complexity, starting with coarse distinctions (e.g., active vs. inactive) that subdivide into more resolved categories (e.g., active subdividing into different types of activity) (Figure 2; e.g., McClune et al., 2014). This allows for a tree that can be easily trimmed post calibration to match the ecological question being studied that has an accompanying accuracy for each trimming. For example, if a study is only interested in when the animal is vigilant versus active when out of the nest, the tree can be trimmed to four behavioral states (moving/not moving) with the knowledge that the accuracy is 90.3%. Second, this provides an opportunity to classify each step separately starting with the longest duration behavior for which the time alignment issue should be trivial, and proceeding toward the shortest duration behavior. Data can be cleaned at each stage ensuring that when distinguishing the shortest duration behavior, the training dataset has the lowest error due to time alignment.
4. Select sample windows based on the duration characteristics of the behavior, and the recording frequency: Sample windows must be large enough to contain multiple samples of acceleration in order to calculate the summary statistics. Generally, calibrations of high frequency recordings use sample windows of 1–2 s (20–80 samples, e.g., Pagano et al., 2017). Using lower recording frequencies require larger windows, and thus run the risk of extending beyond the natural duration of the behavior being classified. Although, it may always be possible to find a summary statistic that can separate two behavioral states at any sample window (Figure 4), selecting an inappropriate window will result in unrealistic behavioral durations (Table 2) leading to biased time budgets (Robson & Mansfield, 2014). Thus, it is critical that careful consideration is given to sample window size, and it may be necessary to incorporate different sized windows for different behavioral categories into the classification, as we did in this study (Figure 2).
5. Select summary statistics that are consistent across individuals: Variation in the placement of accelerometer tags during attachment to each individual may influence deployment angles and what each axis is actually measuring. In our study, despite the fact that all tags were attached in the same orientation on all individuals, the nature and weight balance of the collars resulted in the devices spinning around the neck of the animal and resting in unique orientations for each individual. One option to counteract this problem during calibration is to do individual‐specific calibrations, when possible. The other option is to carefully select summary statistics for the calibration that will not be influenced by this issue, such as statistics that are summaries of all three axes (e.g., ODBA, ΔDBA, or single‐axis values that are not affected by the possible range in deployment angles (e.g., surge axis in our study).

Accelerometers provide an unprecedented potential for ecologists to estimate time‐ and energy‐budgets of many species at a level of detail that is not achievable by traditional methods. We found that the limitations in the applicability of these devices on small species can be alleviated through low frequency recordings without loss in accuracy, though low sampling rates do preclude the detection of very short behavior. With an ability to record behavior continuously on small species regardless of light or weather conditions, ecologists can now not only explore time budgets at seasonal scales as we did here (Figure 5), but also how the timing of behavior is structured throughout a day (Ropert‐Coudert et al., 2004). Having access to this detail provides a means for easily incorporating behavioral responses of species to their environments into broader and more complex questions about how they may interact with the species around them in a changing world.

## CONFLICT OF INTEREST

None declared.

## AUTHORS CONTRIBUTIONS

EKS, ML‐C, MMH, and SB conceived the ideas and designed methodology; EKS and AKM collected the data; EKS analyzed the data and led the writing. All authors contributed critically to the ideas and drafts and gave final approval for publication.

## DATA ACCESSIBILITY

All data from this study are archived on Dryad (https://doi.org/10.5061/dryad.1s1m8r7).
