# Supplementary material for: Behavioral classification of low‐frequency acceleration and temperature data from a free‐ranging small mammal
Source: Ecol Evol. 2018 Dec 27;9(1):619–30. doi: 10.1002/ece3.4786 (PMC6342100; doi:10.1002/ece3.4786)
Supplement: Supplementary file 1 [file ECE3-9-619-s001.docx]

**Supporting Information**

- 1. **Collar Construction Method**

The accelerometer units that we used came unpackaged so that we could construct collars to meet the specifications needed for the small size of the red squirrels. Below are the steps that were taken to protect the accelerometers from claws and teeth of the squirrels as well as the elements. The circuit boards came coated with a protective layer such as plastidip which we then labelled externally (2). We then added a layer of seran wrap (3) and candle wax (4) to protect against moisture. On top of that we constructed a hard casing using a strip from an aluminum drinking can (5). This was formed into a round housing and held in place around the accelerometer with electrical tape (6). Two circular aluminum end pieces were fashioned from the same can and attached with duct tape (7). Care was taken to ensure that all devices were in the same orientation on the animal, so as the device was sealed in the protective housing, the orientation was marked on the housing (8).

We used zip ties for the band of the collar. To give reference to the circumference of the collar when attaching, we mark the zip ties at 9 cm and 10 cm prior to construction (9). These are used when collaring the animal to ensure that no collar is attached too tight. To protect the neck of the squirrel from the rough edge of the zip tie, we coat the zip tie in two lengths of 1/8” heat shrink where the accelerometer will not be placed (10). To attach the accelerometer to the zip tie, we first used a piece of electrical tape (11), then heat shrink. A trapezoidal piece of ¾” heat shrink was used where the short side is the length of the accelerometer and the long side is 1.5X the length of the accelerometer (12). This is centered over the collar band and accelerometer with the short side lying against the band (13). When shrunk with heat, the long side of the heat shrink will fold up and around the base of the accelerometer (14) ensuring that it is securely attached to the collar.


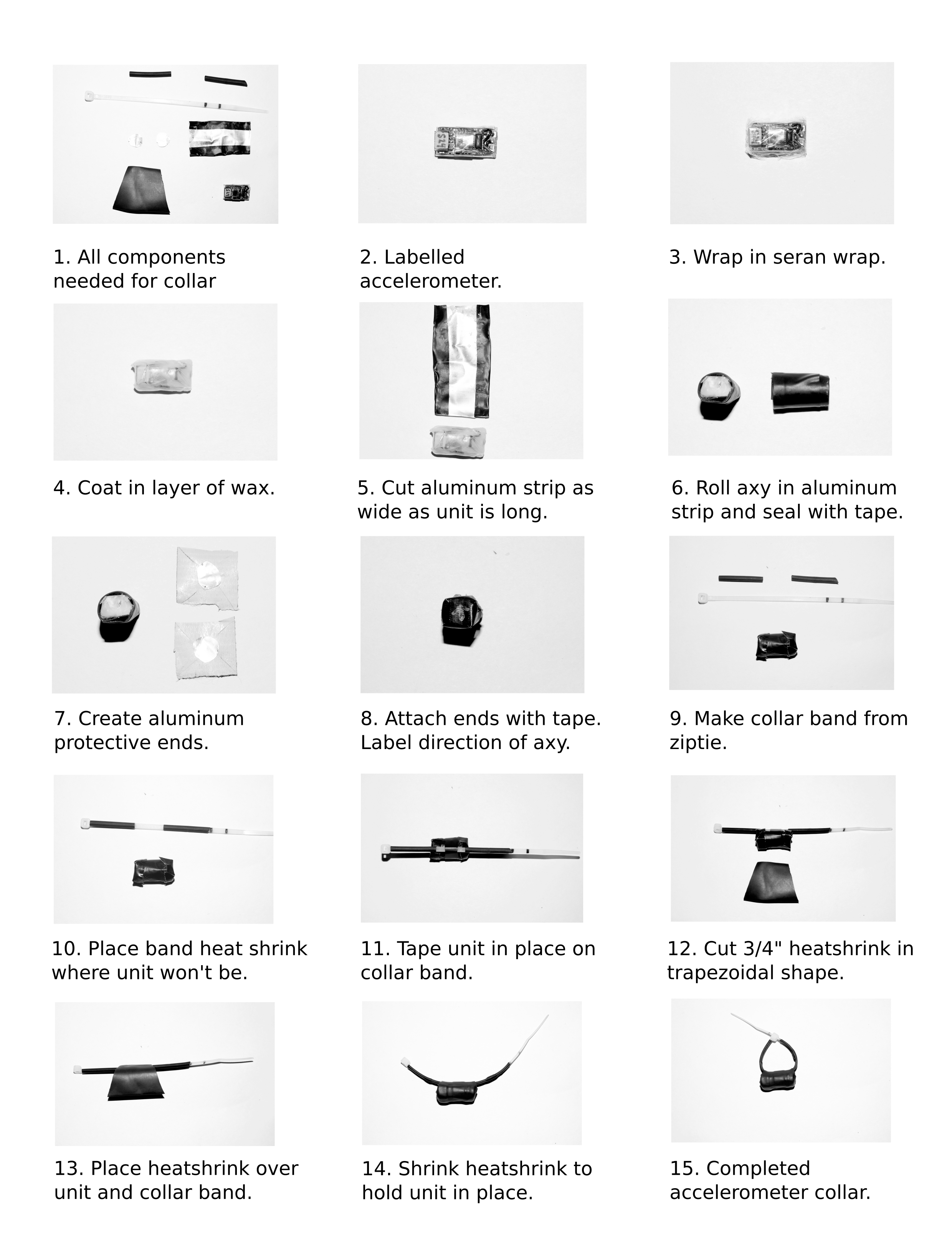


- 1. **Red Squirrel Behavior Data Collection Application**

*This application was designed and developed in 2014 to record instantaneous changes in behavior of wild red squirrels. This app was developed for IOS version 6.1.*

**The Application**

The application consists of two screens: the first collects basic information about the individual that is being viewed and their initial behavior at first observation, the second allows the user to collect instantaneous behavior of the individual with a series of buttons. This saves all the data into 2 .csv files, one for each screen.

*Screen 1:*

OBS – Initials of the observer

Mode – Type of observation being made. On the red squirrel project, we distinguish between casual observations and schedule focal observations.

Colours – Identification markings of the individual. In red squirrels a unique combination of colored ear tags is used for identification.

Sex – Male or Female

Behaviour – a drop down list of behaviours appears to choose from including behaviours like feeding, travelling, and vocalizing.


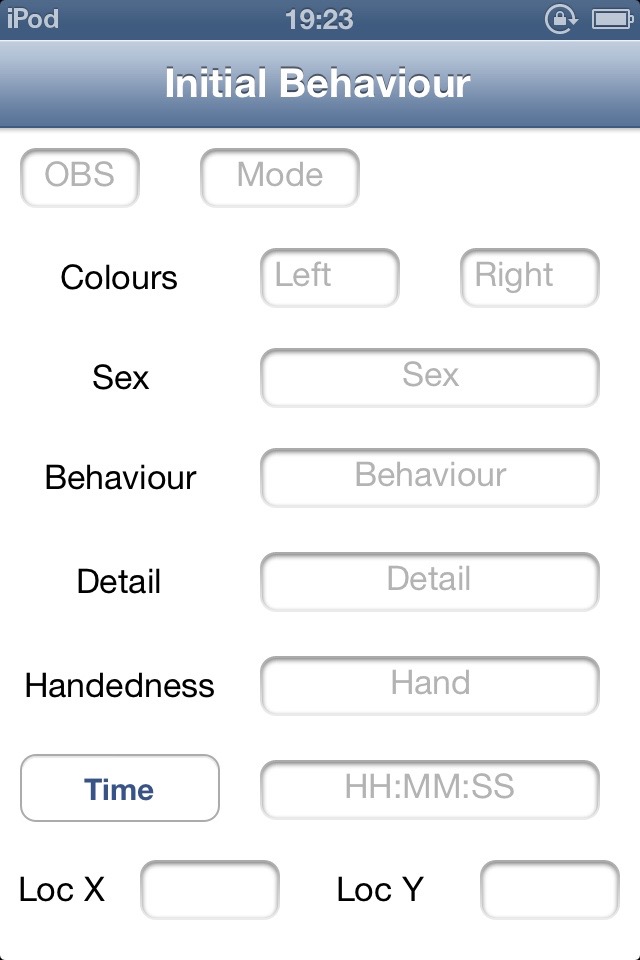
Detail – for some behaviors there are additional details to be filled in. For example, if the animal is feeding, what is being consumed would be recorded in this variable.

Handedness – if the squirrel is feeding, we record whether it is using its left or right forepaw predominantly.

Time – time of observation. If button “Time” is pressed, the current time is filled in.


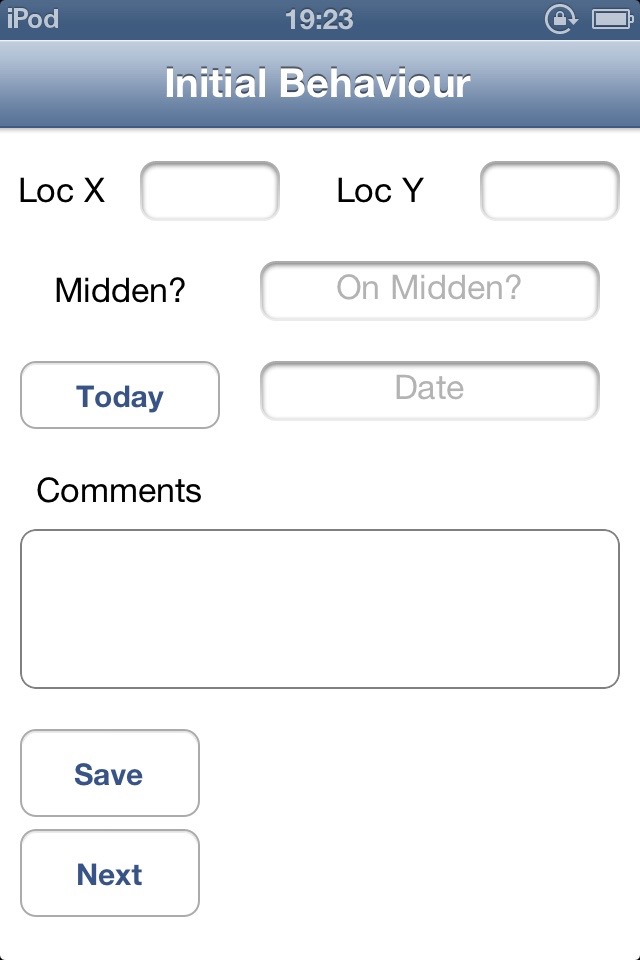
Loc X, Loc Y – the coordinates (X,Y) of the squirrel within the study grid.

Midden? – recorded whether or not the squirrel is on the center larder hoard within its territory.

Date – record the date of the observation. If button “Today” is pressed, the current date will be filled in automatically.

Comments – any additional comments can be recorded here.

Save – press this button to save the observation.

Next – press this button to proceed to the next screen without saving. Information from this screen such as observer, colours, and sex are transferred to the next screen and included in all data saved when on the next screen


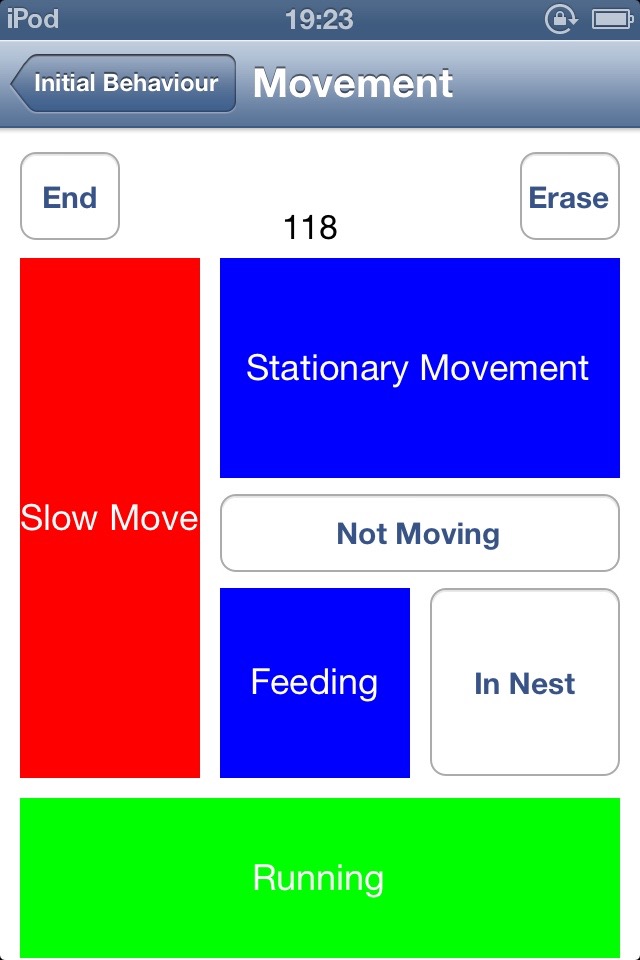
*Screen 2:*

This screen collects instantaneous behaviour of the squirrel. It consists of 6 buttons that each represent a behavioral state. For the red squirrels, these behaviours included slow and fast travel (Slow Move, and Running), Stationary Movement (not moving in space, but body still moving including grooming and vocalizing), Not Moving, Feeding (actively consuming resources), and In Nest (not visible but telemetry signal triangulated to a squirrel nest).

Depending on the common duration of the behavior, the buttons had different functions when tapped. For behavioral states that tended to last longer than 10 secs at a time (Not Moving, In Nest, and Feeding), the application would record one line when the button is tapped indicating the start of the behavioral state. This requires that another button is pressed when the squirrel changes behavioral state to indicate the end of that state.

For behavioural states that typically occur at durations less than 10 seconds (Slow Move, Running, and Stationary Movement), two lines of data will be recorded when the button is pressed. The first when the button is pressed indicating the start of the behavior. The second when the button is released indicating the end of the behavior. For these behavioural states, user must press and hold the button for the duration of the behavior.

Additional functions of this screen include an “End” button that records a line of data indicating the end of the observational period. An “Erase” button that records a line indicating that the previous line of data was a mistake and should be deleted during post observation. There is a timer in the top center of the screen that counts down from 120 seconds to 0 indicating when to finish the observational period.

**The Data**

This application creates two data frames for different purposes. The first (table 1) creates a list of behavioral observations in the standard format used for the long term Kluane Red Squirrel Project. The second was the continuous observations used for this study. This data frame consisted of the observer, squirrel identification, sex, time, behavior, and whether it was when the behavior started or stopped, or the end of the observational period.

Table 1. Example of the data frame that is produced by screen 1 of the application.

| id | obs | mode | lcolor | rcolor | sex | behavior | detail | hand | time | locx | locy | midden | date | com |
| --- | --- | --- | --- | --- | --- | --- | --- | --- | --- | --- | --- | --- | --- | --- |
| 13 | EKS | adfoc | G! | P! | M | In nest |  |  | 10:16:45 | J.7 | 18.9 | On | 2014-02-19 |  |
| 14 | EKS | adfoc | R! | - | M | Travel | Tree |  | 12:03:09 | E.7 | 10.0 | Off | 2014-02-19 |  |
| 15 | EKS | adfoc | Y! | B! | M | Vigilant |  |  | 12:14:42 | C.3 | 13.1 | Off | 2014-02-19 |  |
| 16 | EKS | adfoc | W! | Y! | M | Vigilant |  |  | 12:18:04 | C.0 | 11.2 | On | 2014-02-19 |  |
| 17 | EKS | adfoc | B! | Y! | M | Feeding | Cone | R | 12:26:21 | J.2 | 9.6 | On | 2014-02-19 |  |
| 18 | EKS | adfoc | W! | O! | M | Vocal | rattle |  | 12:34:12 | J.7 | 6.6 | Off | 2014-02-19 |  |
| 19 | EKS | adfoc | O! | P! | M | feeding | mush |  | 12:40:39 | D.8 | 3.1 | on | 2014-02-19 |  |

Table 2. Example of the data frame that is produced by screen 2 of the application.

| ID | OBS | LCOLOUR | RCOLOUR | SEX | TIME | BEHAV | B2 |
| --- | --- | --- | --- | --- | --- | --- | --- |
| 49 | EKS | Y! | B! | M | 2014-03-20 11:03:17 | Nest | PrStart |
| 50 | EKS | Y! | B! | M | 2014-03-20 11:04:18 | END | END |
| 51 | EKS | P! | W! | M | 2014-03-20 11:24:20 | Nest | PrStart |
| 52 | EKS | P! | W! | M | 2014-03-20 11:25:22 | END | End |
| 53 | EKS | W! | Y! | M | 2014-03-20 11:35:15 | StatMove | Start |
| 54 | EKS | W! | Y! | M | 2014-03-20 11:35:21 | StatMove | Stop |
| 55 | EKS | W! | Y! | M | 2014-03-20 11:35:27 | RunningMove | Start |
| 56 | EKS | W! | Y! | M | 2014-03-20 11:35:37 | RunningMove | Stop |
| 57 | EKS | W! | Y! | M | 2014-03-20 11:35:59 | StatMove | Start |
| 58 | EKS | W! | Y! | M | 2014-03-20 11:36:00 | StatMove | Stop |
| 59 | EKS | W! | Y! | M | 2014-03-20 11:36:01 | notmoving | PrStart |
| 60 | EKS | W! | Y! | M | 2014-03-20 11:36:04 | StatMove | Start |
| 61 | EKS | W! | Y! | M | 2014-03-20 11:36:04 | StatMove | Stop |
| 62 | EKS | W! | Y! | M | 2014-03-20 11:36:05 | Feed | Start |
| 63 | EKS | W! | Y! | M | 2014-03-20 11:37:01 | Feed | Stop |
| 64 | EKS | W! | Y! | M | 2014-03-20 11:37:02 | END | END |

- 1. **Time alignment**

To align the clocks on the accelerometers with that on the behavioral observation, it was necessary to visually inspect the acceleration recording in and around the time of the observations to determine the extent of this mismatch. These time discrepancies appeared to be unique to each combination of squirrel, observer, and date. To determine the mismatch, we first identified in the behavioral observations clear transitions from substantial time (>10 sec) being spent not moving to moving as these transitions would generate a clear signal on the accelerometer. From this we were able to calculate the time mismatch for 47 and 13 observer squirrel days in the winter and autumn observation periods, respectively. Sample sizes were smaller in the autumn because at this time squirrels spent very little time not moving and large amounts of time running in comparison to the winter. Accordingly, we additionally identified in the autumnal data transitions from feeding (>30 sec) to fast travel (>7 sec) for additional calculations of time mismatch. From these additional transitions, we calculated the mismatch for a total of 39 observer squirrel days in the autumn. In total there were 11 observer squirrel days with multiple transitions on which a time mismatch could be calculated. In all cases the time mismatch tended to be within 2 seconds of the other transitions with the largest discrepancies being 8 seconds. In the situations where there was more than one transition, we used the mean time mismatch of all transitions for adjusting the time of the observations.

- 1. **Temperature Filtering**

The temperature data loggers had a tendency to sporadically glitch throughout the recording. These glitches appeared to record a temperature that was considerably different (up to 10°C) from the 10 seconds before and after and would record the same temperature at each glitch. Thus, prior to any analysis, we filtered for these by determining the largest temperature difference that can occur naturally from one recording to the next, which is dependent on the temperature differential between in and out of the nest. Using temperature loggers (winter: n=10; autumn: n=10) that were visually confirmed to be functioning correctly over time, we calculated the maximum rate of temperature change from one recording to the next over 4 days (Table 1). Autumn recordings had a max rate of temperature change of -1.22°C and 1.10°C, and winter temperature differences maxed out at -4.40°C and 1.35°C. We identified any temperature recordings that had rates of change greater than 1.5°C per 10 seconds or 5°C per 10 seconds in autumn and winter, respectively. These temperature recordings were replaced by the mean temperature of the preceding and subsequent temperature recordings.

Table 1. Maximum temperature change between two temperature recordings on data loggers deployed on red squirrels in the winter (ambient temperature <0°C) and autumn (ambient temperature >0°C). This rate of change is influenced by the temperature differential between the nest and ambient temperature.

| **Accelerometer** | **MaxTempDiff (neg)** | **MaxTempDiff (pos)** | **Season** |
| --- | --- | --- | --- |
| DD_Mar27_2014 | -1.35 | 1.22 | Winter |
| HH_Sep28_2014 | -0.86 | 0.61 | Autumn |
| TT_Sept24_2014 | -0.86 | 0.86 | Autumn |
| BB_Sep16_2014 | -1.22 | 1.10 | Autumn |
| NN_Sep28_2014 | -1.1 | 0.98 | Autumn |
| UU_Sept24_2013 | -1.1 | 1.1 | Autumn |
| ZZ_Sep28_2014 | -1.22 | 1.1 | Autumn |
| XX_Sept27_2014 | -1.22 | 1.1 | Autumn |
| AF_Sep29_2014 | -1.22 | 0.98 | Autumn |
| SS_Sep28_2014 | -1.10 | 1.10 | Autumn |
| DD_Sept25_2014 | -1.1 | 0.86 | Autumn |
| XX_Feb27_14 | -1.83 | 1.22 | Winter |
| OO_Mar2_14 | -2.57 | 0.74 | Winter |
| CC_mar4_2014 | -4.4 | 1.22 | Winter |
| YY_March4_2014 | -2.81 | 0.98 | Winter |
| II_Mar29_2014 | -1.34 | 0.86 | Winter |
| DD_Mar13_2014 | -2.93 | 1.35 | Winter |
| EE_Mar17_2014 | -2.32 | 0.86 | Winter |
| BB_Mar29_2014 | -1.96 | 0.98 | Winter |
| CC_mar4_2014 | -4.4 | 1.22 | Winter |

- 1. **Running Means Smoothing Window for Static Acceleration**

To separate static acceleration from dynamic acceleration, a running means smoothing window is applied to the raw acceleration. The smoothed file is the static acceleration. Dynamic acceleration is calculated by removing the static acceleration from the raw acceleration. Selection of the window size over which the smoothing function is important as too short a window may result in much of the dynamic acceleration being captured as static. This can cause implications on the summary statistics like ODBA that are derived from the dynamic acceleration. To determine if our selected window size (91 s) was appropriate we followed recommendations proposed by Shepard et al. (2008) and completed a sensitivity analysis by calculating ODBA using dynamic acceleration generated from running mean smoothing functions with windows ranging from 3 s to 220 s (Figure 1). For this we selected accelerometer files from 8 red squirrels and from each file we randomly selected one complete day of data producing 8 days of acceleration. As ODBA for different behavioral states may stabilize at different smoothing function window size (Shepard et al., 2008), we completed this sensitivity analysis for three different behavioral states: running (Figure 1a), foraging (Figure 1b), and feeding (Figure 1c). We confirmed that our window size (91s) was above the point at which ODBA became less variable with changes in the length of the running mean.

Shepard, E., Wilson, R., Halsey, L., Quintana, F., Gómez Laich, A., Gleiss, A., … Norman, B. (2008). Derivation of body motion via appropriate smoothing of acceleration data. *Aquatic Biology*, *4*(3), 235–241. doi:10.3354/ab00104


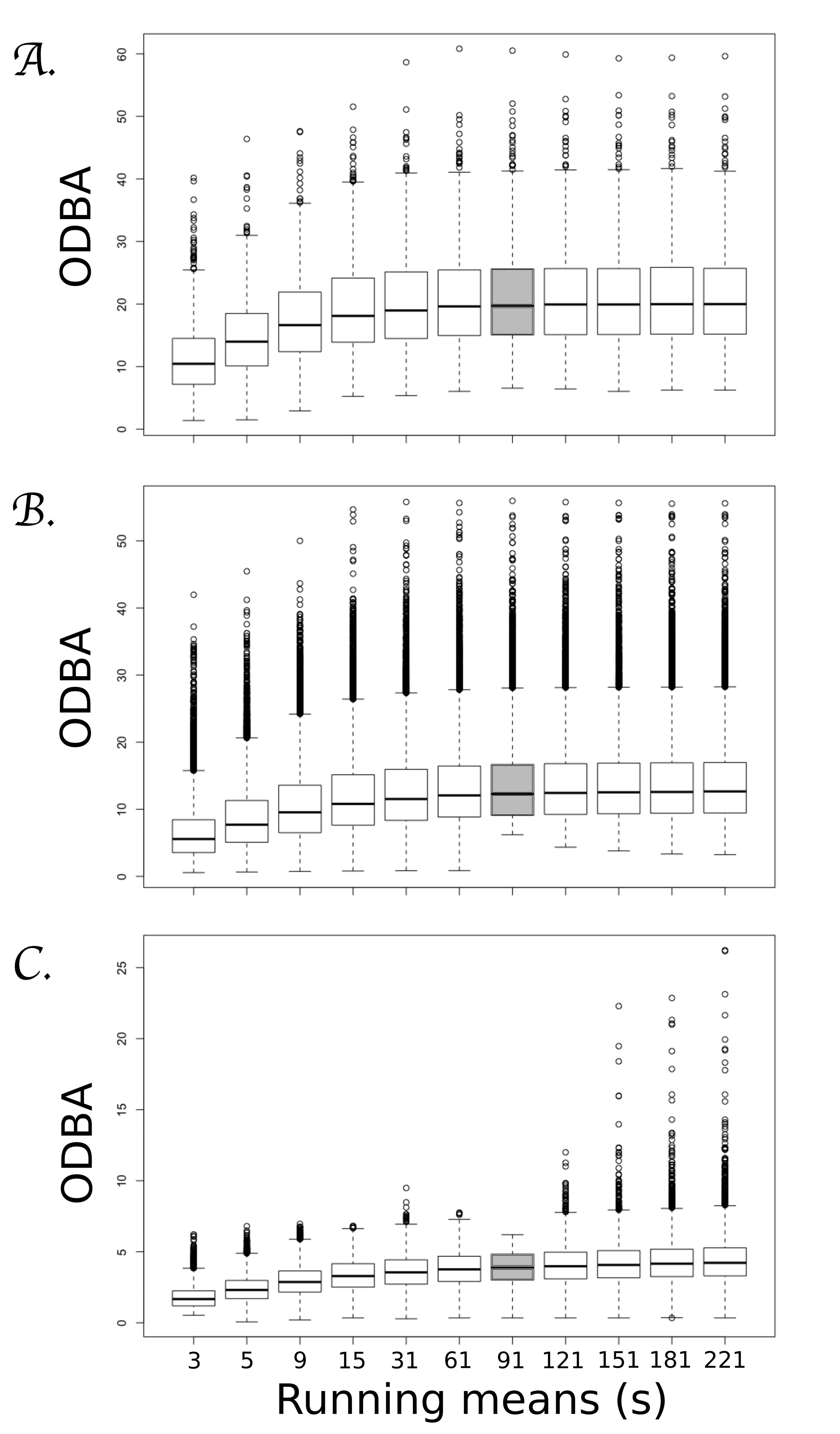


Figure 1. Change in overall dynamic body acceleration (ODBA; median and 25^th^/75^th^ quantiles) as acceleration data is smoothed at increasing running means window sizes from 3 s to 221 s for running (A), foraging (B), and feeding (C) behavioral states over 1 day in 8 red squirrels. Grey box (91 s) represents the window size selected for calibration of red squirrel accelerometer data.
